# The Association of Vitamin D during Pregnancy and mRNA Expression Levels of Inflammatory Factors with Preterm Birth and Prelabor Rupture of Membranes

**DOI:** 10.3390/nu15153423

**Published:** 2023-08-02

**Authors:** Xialidan Alifu, Shuting Si, Yiwen Qiu, Haoyue Cheng, Ye Huang, Peihan Chi, Yan Zhuang, Haibo Zhou, Libi Zhang, Diliyaer Ainiwan, Zhicheng Peng, Hui Liu, Yunxian Yu

**Affiliations:** 1 Department of Public Health, and Department of Anesthesiology, Second Affiliated Hospital of Zhejiang University School of Medicine, Hangzhou 310009, China; 3130100017@zju.edu.cn (X.A.); shutingsi@zju.edu.cn (S.S.); yiwenqiu@zju.edu.cn (Y.Q.); 3150101365@zju.edu.cn (H.C.); 22218854@zju.edu.cn (Y.H.); 22118872@zju.edu.cn (P.C.); yanzhuang@zju.edu.cn (Y.Z.); 11918158@zju.edu.cn (H.Z.); 15313710752@163.com (L.Z.); 22218236@zju.edu.cn (D.A.); 22018678@zju.edu.cn (Z.P.); 2 Department of Epidemiology & Health Statistics, School of Public Health, School of Medicine, Zhejiang University, Hangzhou 310058, China; 3 Yiwu Maternity and Children Hospital, Yiwu 322000, China; 4 Sir Run Run Shaw Hospital, School of Medicine, Zhejiang University, Hangzhou 310058, China; lhui2010@zju.edu.cn

**Keywords:** vitamin D, PTB, TPROM, PPROM, mechanism

## Abstract

The aim of this study was to elucidate the association between vitamin D (VD) and the risk for preterm birth (PTB) and prelabor rupture of membranes (PROM). This study included two parts, with a cohort study and a case-control study. Plasma 25-hydroxyvitamin vitamin D [25(OH)D] levels in three trimesters in the cohort study and maternal 25(OH)D before delivery in the case-control study were measured. Quantitative real-time PCR was used to detect relative mRNA expression levels of the inflammatory factors associated with pyroptosis in peripheral blood mononuclear cell (PBMC), placenta and fetal membranes. Multinomial logistic regression and the Wilcoxon test were applied to analyze the associations. In the cohort study, 6381 pregnant women were included. We found that VD deficiency in T3 (PTB without PROM: OR = 1.90, 95% CI: 1.02–3.55, Term PROM (TPROM): OR = 0.76, 95% CI: 0.59–0.98) and less change of 25(OH)D between T1 and T3 (PTB without PROM: OR = 2.32, 95% CI: 1.07–5.06, TPROM: OR = 0.73, 95% CI: 0.56–0.96) were associated with the increased risk of PTB without PROM, while there was a decreased risk of TPROM. Neither VD, nor the increase of VD during pregnancy was associated with the premature rupture of membranes preterm delivery (PPROM). In the case-control study, there were no associations between VD during delivery and PTB or PROM (TPROM: OR = 1.33, 95% CI: 0.52–3.44); PTB without PROM: OR = 1.66, 95% CI: 0.33–8.19; PPROM: OR = 1.19, 95% CI: 0.42–3.40). The mRNA expression of NLRP1 (NOD-like receptor thermal protein domain associated protein 1) (*p* = 0.0165) in PBMC in the TPROM group was higher than that in the term group, and IL-18 (*p* = 0.0064) was lower than that in the term group. Plasma 25(OH)D in T3 and the increase of 25(OH)D between T1 and T3 were associated with a lower risk for PTB without PROM but a higher risk for TPROM. Further studies are warranted to clarify the association between VD and PTB and PROM and its mechanism.

## 1. Introduction

Preterm birth (PTB) is defined as delivery before 37 weeks of pregnancy. The incidence of PTB ranges from 5% to 18% worldwide [1]. In recent years, the incidence of PTB in China has been reported to be approximately 5.0%, but the total number of premature babies in China ranks second in the world; it is only less than that in India [2,3,4,5]. PTB complications are the major cause of death in newborns and children under 5 years of age [6]. However, the etiology of PTB is complex and the mechanism is unknown. Therefore, it is still of great public health significance to explore effective preventive measures against PTB. Clarifying the pathogenesis of PTB and seeking prevention methods have been one of the key issues of maternal and child health. Premature rupture of membranes (PROM) refers to the rupture of the membranes before delivery. The premature rupture of membranes occurring before and after 37 weeks of gestation are called preterm premature rupture of membrane (PPROM) and term premature rupture of membrane (TPROM), respectively [7]. The reported incidence rates of TPROM and PPROM in China were 12.1–15.6% and 2.0–3.1%, respectively [8,9,10,11]. PTB and PROM are both associated with inflammation. 

Vitamin D (VD) has the function of regulating the immune response, so we speculate that VD may be mainly related to the occurrence of PTB and PROM. However, VD deficiency was common in all kinds of people, especially in pregnant women [12]. In 2017, a meta-analysis reported that VD deficiency during pregnancy was related to an increased risk of PTB, but was not associated with the risk of spontaneous PTB [13]. However, in 2018, a meta-analysis of the Spanish cohort did not find that VD levels were associated with PTB [9]. The study from Singapore also found no correlation between VD deficiency and PTB [10]. The study from Guangzhou even showed the opposite finding that VD deficiency was associated with a higher risk of PTB [11]. Therefore, the association between VD and PTB is still contradictory. Furthermore, to our knowledge, there are few studies that have reported on the association between VD and PROM.

Pyroptosis is a pro-inflammatory programmed cell death mediated by cysteine aspartic protease 1 (Caspase-1) dependence, accompanied by the release of a large number of pro-inflammatory factors, and induces a series of amplified inflammatory reactions. Toll-like receptors (TLRs) stimulate the production of inflammatory cytokine precursors (Pro-IL-1β, Pro-IL-18, etc.) and NLRPs (NOD-like receptor thermal protein domain associated proteins). NLRPs recruit adapter protein (ASC) and Pro-Caspase-1 to form inflammasome, which then activates Pro-Caspase-1 to produce enzymatically active Caspase-1 and process Pro-IL-1β and Pro-IL-18 to IL-1β and IL-18. At the same time, Caspase-1 destroys the integrity of the membrane by cutting the downstream executive protein Gasdermin D, leading to cell lysis, releasing inflammatory factors into the extracellular areas and further expanding the inflammatory response, inducing the pyroptosis of cells [14]. At present, the common inflammasomes involved in the pyroptosis of macrophages include NLRP3, NLRP1, NLRC4, etc. [15]. Induced by lipopolysaccharide, human monocytes and macrophages can produce cytokines, such as TNF-α, IL-1β, IL-6, IL-8, and IL-10 [16]. Hence, we speculated that macrophages might activate Caspase-1 by activating inflammasome, generate inflammatory factors, and induce cell pyroptosis, further leading to PTB and PROM. VD has anti-inflammatory effects and can regulate a variety of immune cells (such as monocytes, macrophages, B cells, and T cells) to reduce the production of pro-inflammatory factors and increase the level of anti-inflammatory factors [17]. Experiments in mice have shown that prenatal vitamin D supplementation leads to a decrease in the number of macrophages in the lungs of newborns [18]. Cell experiments showed that mRNA expression levels of IL-1β were reduced in 1,25(OH)_2_D_3_-treated uterine myocytes [19]. Based on these, we assumed that VD might be involved in the occurrence of PTB and PROM by regulating pyroptosis. However, no relevant studies have been reported.

Therefore, the aim of this study was to elucidate the association of VD in different trimesters with PTB and PROM in the cohort study. The expression levels of plasma inflammatory factors in peripheral blood mononuclear cells (PBMC), fetal membranes and placental tissues of pregnant women were detected in the case-control study to explore the molecular mechanism of VD regulating pyroptosis in PTB and PROM.

## 2. Materials and Methods

### 2.1. Study Design and Population

This study included two parts: the cohort study and case control study. First, the cohort study was from a running Zhoushan Pregnant Women Cohort that had been established since August 2011 in Zhoushan Maternal and Child Health Care Hospital, Zhejiang Province. Pregnant women are recruited at the first trimester, according to the inclusion criteria and exclusion criteria. This study included the participants who were recruited between August 2011 and June 2022. The detailed criteria have been described in a previous study based on this cohort [20]. In the cohort, pregnant women between 18 and 45 years old, who had their first visit between 8 and 14 gestational weeks (first trimester, T1), who were without severe physical, mental illness, threatened abortion, fetal abnormalities, and who received at least one time 25(OH)D concentration detection were included in this manuscript. Second, in order to collect PBMC to further explore the molecular mechanism, the case-control study was conducted. We recruited pregnant women with PPROM, PTB, and TPROM and the corresponding term control, who were admitted to the hospital expecting to give birth between June and September in 2021 and 2022 at Yiwu Maternity and Child Health Care Hospital and in 2022 at Zhoushan Maternal and Child Health Care Hospital. Finally, 37 cases of PPROM and 37 corresponding term mothers were taken as control 1, 16 cases of preterm without PROM and 15 corresponding term mothers were taken as control 2, and 58 cases of TPROM and the controls (control 1 and control 2) were included (Supplementary Figure S1). Informed consent was obtained from all participants before they were included. The study protocol was approved by the ethics board of Zhejiang University School of Medicine (No. 2019-067).

### 2.2. Information and Sample Collection 

#### 2.2.1. Cohort Study

A baseline survey was conducted after being included in the cohort between 8 and 14 gestational weeks by a trained interviewer in the form of face-to-face interviews with pregnant women. The information of general sociodemographic characteristics, lifestyle behavior habits, dietary habits, nutritional status, reproductive and marital history, previous history of adverse pregnancy, previous complications, drug use during pregnancy, early pregnancy reaction (nausea and vomiting, chest pain, and the increase of leukorrhea) etc. were collected. Follow-up was conducted at 24–28 gestational weeks (second trimester, T2) and 32–36 gestational weeks (second trimester, T3) and relevant information of the subjects during the two follow-up periods was collected, including lifestyle, behavior, diet, nutrition, and medication. On the day of the epidemiological survey in T1, T2, and T3, 5 mL of peripheral venous blood was collected from each subject and were stored under −80 °C until use.

#### 2.2.2. Case-Control Study

With the informed consent of the subjects, 5 mL of peripheral venous blood was collected using a vacuum collection vessel containing EDTA, and peripheral blood mononuclear cells were extracted immediately after collection. The basic information of the pregnant women was extracted from the Electronic Medical Records System (EMRS), including age, pre-pregnancy BMI, gravidity, parity, the history of delivery week and delivery mode. Within 5 min after the delivery of the placenta by caesarean section or vaginal delivery, the placenta tissue (including fetal and maternal surfaces) with an area of approximately 3 × 3 cm^2^ (including fetal and maternal surfaces) should be removed longitudinally from the maternal surface within a range of 4–5 cm around the junction of the placenta and umbilical cord. The placental calcification and necrosis foci, such as fascia, should be avoid. After being fully rinsed with 4 °C DEPC water, the fetal membrane was separated from the surface of the placenta. Approximately 1.5 g of fetal membrane tissue was clipped, and the tissue was frozen in liquid nitrogen for 15 min, then removed and stored in a −80 °C refrigerator.

### 2.3. Measurement of 25(OH)D Concentrations

Plasma 25(OH)D_2_ and 25(OH)D_3_ concentrations were measured with liquid chromatography tandem-mass spectrometry (API 3200MD (Applied Biosystems/MDS Sciex, Waltham, MA, USA)). The plasma 25(OH)D concentration was reported in ng/mL, which was the sum of 25(OH)D_2_ and 25(OH)D_3_. The minimum sensitivity of the measurement was 2 ng/mL for 25(OH)D_2_ and 5 ng/mL for 25(OH)D_3_. The intra-assay coefficients variances (CVs) of 25(OH)D_2_ and 25(OH)D_3_ were 1.47~7.24% and 2.50~7.59%, respectively. The inter-assay CVs were 4.48~6.74% and 4.44~6.76% for 25(OH)D_2_ and 25(OH)D_3_, respectively [20].

### 2.4. Measurement of mRNA Expression Levels of Inflammasome Related Molecules

We conducted quantitative real-time PCR to detect relative mRNA expression levels. The total RNA was extracted from the placenta and fetal membranes and the peripheral blood mononuclear cell (PBMC), respectively, and reverse-transcribed into cDNA using PrimeScript RT Master Mix (RR036A, Takara). Primers (Supplementary Table S1) were designed using PrimerBank (https://pga.mgh.harvard.edu/primerbank/ (accessed on 17 December 2021)). The reaction was performed in a Roche LightCycler^®^ 480 (Basel, Switzerland) with a quantification using a TB Green dye method test kit (RR420A, Takara, San Jose, CA, USA). The housekeeping gene *GAPDH* was an internal control, and we adopted the 2^−ΔΔCt^ method to analyze the relative gene expression.

### 2.5. Variable Definition

We defined VD deficiency as 25(OH)D < 20 ng/mL, according to the Endocrine Society Clinical Practice Guideline [21]. The change of 25(OH)D was the difference of 25(OH)D levels between trimesters (back minus front) and was categorized into two groups, according to the median of corresponding change levels. We divided the seasons into two groups: summer/autumn (June to November) and winter/spring (December to May), according to the duration and intensity of sunshine. Gestational age was calculated according to the date of last menstruation and confirmed by B-scan ultrasound. BMI at the first visit (cohort) or pre-pregnancy BMI (case-control study) was calculated by the weight in kilograms divided by the square of height in meters. Gravidity was divided into 1 time, 2 times, and ≥3 times, and parity was categorized into 0 and ≥1 times. Education was categorized into primary high school and below, high school, and college and above. Diabetes mellitus (DM) included diabetes mellitus before pregnancy and gestational diabetes mellitus. Hypertension disorder of pregnancy (HDP) included chronic hypertension, gestational hypertension, preeclampsia, and eclampsia. We defined PTB as gestational age < 37 weeks at delivery. According to the practice bulletin of the American College of Obstetrics and Gynecology (American College of Obstetricians and Gynecologists, 2020), prelabor rupture of membranes (PROM) was defined as the outflow of amniotic fluid around the fetus before the start of uterine contraction, and divided into term PROM (TPROM) and preterm PROM (PPROM) [1,2], depending on whether the gestational age of delivery is 37 weeks or greater.

### 2.6. Statistical Analysis

Continuous variables and categorial variables were presented as mean ± SD and frequency (percentage), respectively. Due to the skewed distribution of inflammatory factors, the median and inter-quartile range (IQR) was adopted. To compare the characteristics between groups, one-way analysis of variance/Student’s *t*-test, and the Chi square test were used for continuous variables and categorical variables, respectively. Multinomial logistic regression was conducted to explore the association of 25(OH)D, VD status, and the increase of 25(OH)D with PTB or prelabor rupture of membranes. Model2 were adjusted for BMI at first visit, age, education, parity, and hypertension disorder of pregnancy, and model3 was further adjusted for the gestational age of VD detection, season of VD detection, and delivery mode. The Wilcoxon test was used for the comparison of mRNA expression levels relative to the GAPDH of inflammatory factors between groups. In this study, the level of *p* < 0.05 was considered as statistically significant. All analyses were performed using R software (version 4.0.2) (http://www.R-project.org (accessed on 22 June 2020)).

## 3. Results

### 3.1. Cohort Study

#### 3.1.1. Characteristics of Study Population

In the cohort study, 6381 pregnant women were included. As shown in Table 1, maternal age, BMI at first visit, weight gain, education, parity, HDP, and delivery mode were all significantly different among the groups (*p* < 0.05), but the prevalence of DM was balanced among the groups (*p* = 0.195).

#### 3.1.2. The Association between Maternal Vitamin D Level and PTB or PROM

As shown in Table 2, we only found significant difference in the 25(OH)D levels, gestational age of VD detection, and VD status among term, TPROM, PTB without PROM, and PPROM groups in T3 (*p* < 0.05), while there was no difference in T1 and T2. In terms of the detection season of VD, it was all balanced in the T1, T2, and T3 among the four groups. We also conducted multinomial logistic regression, and found that VD deficiency in T3 was associated with an increased risk of PTB without PROM in model 2 (OR = 1.90, 95% CI: 1.02–3.55). However, VD deficiency in T3 was associated with a decreased risk of TPROM in model 2 (OR = 0.76, 95% CI: 0.59–0.98). In model 3, when further considering gestational age of VD detection, season of VD detection, and delivery model, no statistically significant difference was found (Table 3). Analysis was repeated among those with vaginal delivery to avoid the effect of medical measures on gestational age of delivery and the results were similar (Supplementary Table S2). In terms of VD levels, change between T1 and T3, compared with an increase of 25(OH)D between T1 and T3 of 10.8 ng/mL and more, those with increase of 25(OH)D within 10.8 ng/mL were associated with a lower risk for TPROM (OR = 0.73, 95% CI: 0.56–0.96), but a higher risk for PTB without PROM (OR = 2.32, 95% CI: 1.07–5.06) (Table 4). However, compared with an increase of 25(OH)D between T1 and T2 of 10.4 ng/mL and more, those with increase within 10.4 ng/mL were associated with a lower risk for PTB without PROM in model 3 (OR = 0.58, 95% CI: 0.35–0.98). No significant association was found between a change of 25(OH)D between T2 and T3 and TPROM (OR = 0.77, 95% CI: 0.56–1.05) or PTB without PROM (OR = 2.54, 95% CI: 0.65–9.96) (Supplementary Tables S3 and S4.). Surprisingly, neither VD, nor an increase of VD during pregnancy was associated with PPROM.

### 3.2. Case-Control Study

#### 3.2.1. Characteristics of Study Population

In order to further explore the mechanism of the association of VD with PTB and PROM, we conducted the case-control study. Finally, 37 cases of PPROM and the corresponding 37 term mothers, taken as control 1, 16 cases of preterm without PROM and 15 corresponding term mothers taken as control 2, and 58 cases of TPROM and controls (control 1 and control 2) were included. Gravidity, parity, delivery mode, maternal age, and pre-pregnancy BMI were all balance between PPROM and its control, and preterm without PROM and its control. However, gravidity (*p* = 0.047) and the delivery mode were significantly difference between TPROM and term (*p* = 0.001) (Table 5). 

#### 3.2.2. The Association between Maternal Vitamin D during Delivery and PPROM

The prevalence of VD deficiency in PTB without PROM group was higher than that in term group, but the difference was not statistically significant (25.0% vs. 0%, *p* = 0.101) (Table 5). When we conducted the multinomial logistic regression, pre-pregnancy BMI, age, parity, diabetes, hypertension disorder of pregnancy, delivery mode, and year of VD detection were adjusted in model 2. However, both the crude model and adjusted model showed no association between VD during delivery and PTB or PROM (TPROM: OR = 1.33, 95% CI: 0.52–3.44); PTB without PROM: OR = 1.66, 95% CI: 0.33–8.19; OR = 1.19, 95% CI: 0.42–3.40) (Supplementary Table S5).

#### 3.2.3. Comparison of mRNA Expression of Inflammatory Factors in PBMC, Placenta, and Fetal Membrane between Cases and Corresponding Controls between Groups

**Figure 1:**
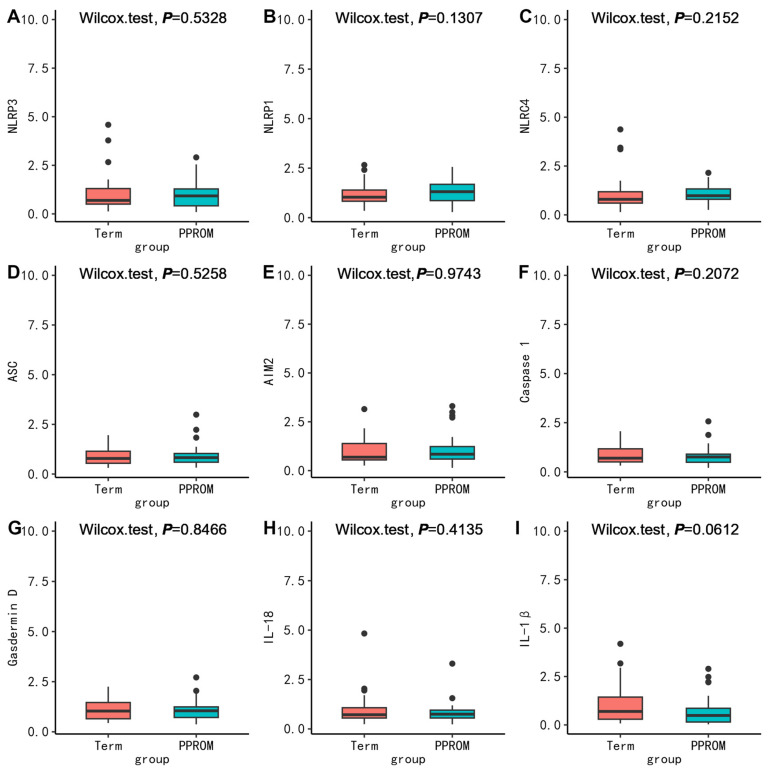
Comparison of mRNA expression levels relative to GAPDH of inflammatory factors (NLRP3 (**A**), NLRP1 (**B**), NLRC4 (**C**), ASC (**D**), AIM2 (**E**), Caspase 1 (**F**), Gasdermin D (**G**), IL-18 (**H**), IL-1β (**I**)) in PBMC between PPROM and term. The Wilcoxon test was used to compare groups. PPROM, preterm prelabor rupture of membranes.

**Figure 2:**
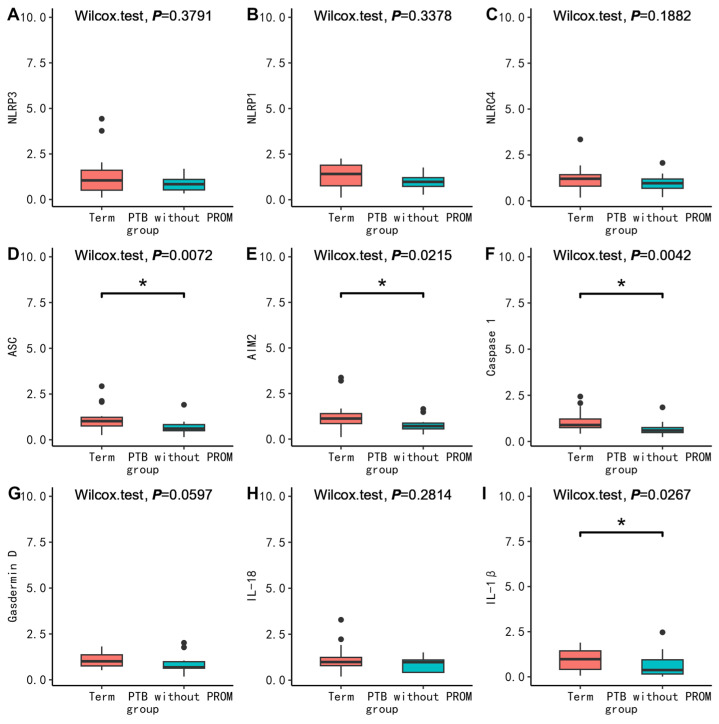
Comparison of mRNA expression levels relative to GAPDH of inflammatory factors (NLRP3 (**A**), NLRP1 (**B**), NLRC4 (**C**), ASC (**D**), AIM2 (**E**), Caspase 1 (**F**), Gasdermin D (**G**), IL-18 (**H**), IL-1β (**I**)) in PBMC between PTB without PROM and term. The Wilcoxon test was used to compare groups. * *p* < 0.05. PTB, preterm birth; PROM, prelabor rupture of membranes.

**Figure 3:**
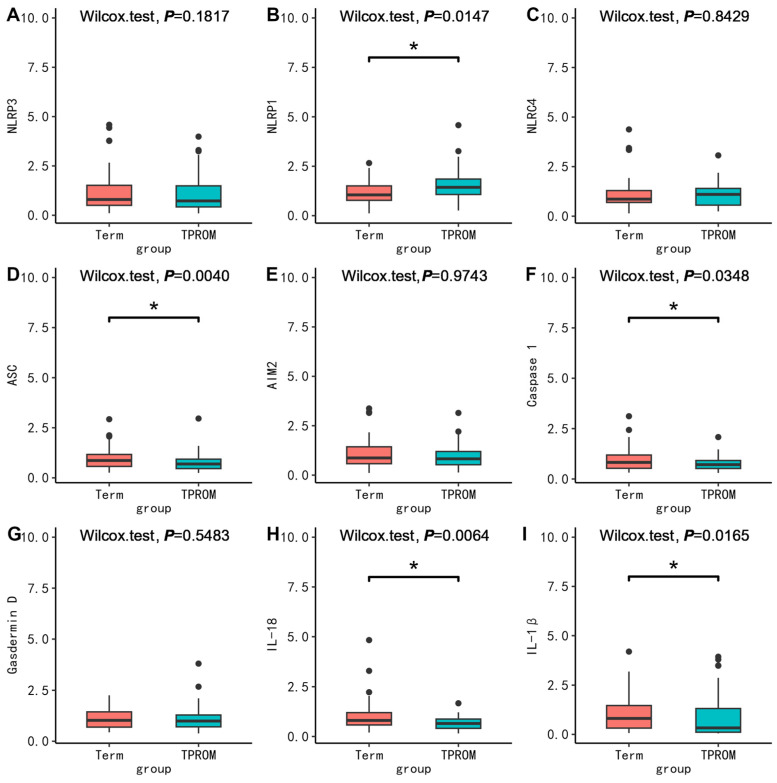
Comparison of mRNA expression levels relative to GAPDH of inflammatory factors (NLRP3 (**A**), NLRP1 (**B**), NLRC4 (**C**), ASC (**D**), AIM2 (**E**), Caspase 1 (**F**), Gasdermin D (**G**), IL-18 (**H**), IL-1β (**I**)) in PBMC between TPROM and term. The Wilcoxon test was used to compare groups. * *p* < 0.05. TPROM, term prelabor rupture of membranes.

To further clarify the possible molecular mechanism, we compared the mRNA expression levels of pyroptosis-related molecules between groups. First, in PBMC, we found no difference in the mRNA expression between PPROM and term (*p* > 0.05) (Figure 1). Surprisingly, the mRNA expression of ASC (*p* = 0.0072), AIM2 (*p* = 0.0215), Casepase-1 (*p* = 0.0042), and IL-1β (*p* = 0.0267) in PBMC in the PTB without PROM group were all lower than that in the term group (Figure 2). The mRNA expression of ASC (*p* = 0.0040), Casepase-1 (*p* = 0.0348), IL-18 (*p* = 0.0064), IL-1β (*p* = 0.0165) in PBMC in the TPROM group were all lower than that in term group (Figure 3). However, the mRNA expression of NLRP1 (*p* = 0.0165) in PBMC in TPROM group was higher than that in term group (Figure 3). Unfortunately, all mRNA expression of inflammatory factors in the placenta and fetal membrane had no significant difference between the groups (Supplementary Tables S6 and S7).

## 4. Discussion

In this study, we found that VD deficiency and a lesser increase of 25(OH)D between T1 and T3 were associated with a higher risk for PTB without PROM but with a lower risk for TPROM. The mRNA expression of NLRP1 in PBMC in the TPROM group was higher than that in term group. However, the mRNA expression of ASC, AIM2, Casepase-1, IL-1β in PBMC in PTB without PROM group, ASC, Casepase-1, IL-18, IL-1β in PBMC in the TPROM group were all lower than that in the term group. Unfortunately, no association was found between VD and PPROM or the mRNA expression of pyroptosis-related factors and PPROM.

In 2022, a meta-analysis including 53 observational studies concluded that maternal 25(OH)D concentration was inversely associated with the risk of PTB (pooled RR = 0.67, 95% CI 0.57–0.79), although there was considerable heterogeneity among the studies (I2 = 79.9%, p heterogeneity < 0.001) [22]. Studies published in 2023 also supported the finding. A nested case-control study in Bangladeshi pregnant women reported that VD deficiency (≤30.25 nmol/L) in T2 was associated with an increased risk for PTB (OR = 1.53, 95% CI: 1.10–2.12) [23]. A study from Taipei found that a daily dose of 2000 IU of VD supplementation (starting at 12–16 gestational weeks) could not only increase the level of 25(OH)D, but also resulted in lower rates of PTB (6.67% vs. 11.19%, *p* = 0.007) [24]. In addition, VD sufficiency was found to modify the association between lead and higher risk for PTB, and spontaneous PTB in cohort studies [25]. In our previous study, we also found that VD levels was inversely associated with the gestational age of delivery [5]. In consistency with the studies above, we also found VD deficiency in T3 and a lesser increase of 25(OH)D between T1 and T3 to be associated with a higher risk for PTB without PROM. During pregnancy, 1,25(OH)2D levels increase twofold [26], mainly due to increased 1α-hydroxylase activity in the maternal kidneys, placenta, and decidua [27]. There are also increases of 1,25(OH)2D from 10 to 12 weeks, peaks in the third trimester, and returns to pre-pregnancy levels after delivery [26]. Therefore, increased VD throughout pregnancy is essential for a normal pregnancy. However, some studies reported no association between VD and PTB [28]. The subgroup analysis of the meta-analysis showed that the association between VD and PTB disappeared when the blood sample type was plasma. In our study, our sample was plasma, which might partly explain why VD in most trimesters was not associated with PTB in our study. Nevertheless, a study from Guangzhou even found that VD sufficiency (≥30 ng/mL) was positively associated with the risk for PTB (OR = 1.039, 95% CI: 1.02–1.06) [29]. The different results might be related to the heterogeneity of the study population, biological samples, inadequate control for confounding, and heterogeneity of exposure measures. Moreover, the optimal threshold for VD during pregnancy remains controversial [30]. In addition, this study showed that VD was only inversely associated with PTB without PROM, which indicated that previously reported differences in the association between VD and PTB might be due to differences in the proportion of different types of PTB. Song et al. [31] also found that VD-binding proteins in cervicovaginal fluid could predict PTB without PROM, but not PPROM. Contrary to our expectations, we found that VD was positively associated with the risk of TPROM. We found no other studies to support this result to now, but we speculated that the opposite direction of the association of VD with PROM and PTB might be one of the reasons for the disappearance of the association between VD and PPROM. 

To validate the findings in the cohort study, we further explored the molecular mechanisms. The regulation of maternal immune function is essential for a successful pregnancy, and a normal pregnancy depends on a balance between immune tolerance and immunosuppression. Any imbalance of pro-inflammatory factors and anti-inflammatory factors can lead to abnormal inflammatory responses, which can lead to adverse pregnancy outcomes such as preterm birth [32]. Existing studies suggested that the level of serum inflammatory factors during pregnancy was associated with PTB. A variety of inflammatory markers, such as IL-1β, granulocyte colony-stimulating factor (G-CSF) and TNF-α were associated with PTB [33,34]. It has been reported that lipopolysaccharide induces the secretion of IL-1β by trophoblastic cells in early pregnancy [35,36]. Animal models of lipopolysaccharide-induced amniotic inflammation showed increased mRNA expression of inflammatory-related genes (*NLRP3*, *Caspase-1*, *IL-1β*), and higher concentrations of NLRP3 protein in fetal membranes and deciduae than before preterm delivery [37]. The inhibition of NLRP3 inflammasomes could prevent PTB [38], and the activation of NLRP1, NLRP3, AIM2, and NLRC4 inflammasomes in PROM was increased [39]. IL-18 may be a defensive cytokine that protects the mother from infection and terminates pregnancy [40]. Similarly, we found that *NLRP1* in the TPROM group was significantly higher than that in the term group, and *IL-18* in the TPROM group was significantly lower than that in the term group. However, in the current study, the mRNA expression of *ASC*, *AIM2*, *Casepase-1*, *IL-1β* in PBMC in PTB without PROM group, and *ASC*, *Casepase-1*, *IL-1β* in PBMC in the TPROM group were all lower than that in the term group. Labor itself is an inflammatory process, and the expressions of *NLRP3* and *ASC* were significantly increased in the chorionic membrane of both full-term and preterm labor [41]. The blood samples in our study were collected when awaiting delivery; most participants with term were even after opening three fingers and having regular contractions appear. Therefore, we speculated that the difference in sampling time might be the reason for the higher levels of inflammatory factors in PBMC in pregnant women with term. Future studies should pay more attention to the confounding caused by sampling time. Unfortunately, we did not find differences about VD before delivery between groups, and did not find differences in cytokines related to pyroptosis in placenta and fetal membrane between groups. Therefore, the association of VD with PTB and PROM and its mechanisms need to be explored in more studies. In addition, future research may better clarify the role of VD in the outcomes by including wider variables with which VD can interact, such as maternal and placental melatonin and cortisol/11B-hydroxysteroid dehydrogenase (HSD)1/2 [42,43]. The inclusion of such factors known to regulate preterm birth as well as maternal, placental, and fetal outcomes should better clarify the role of VD in the outcomes measured in this study and the processes significantly modulated. The aryl hydrocarbon receptor (AhR) is an important regulator of maternal and placental function, with relevance to the outcomes measured in this study [44]. The VD receptor significantly interacted with the AhR, as shown in other cell types [45]. This inclusion of wider regulatory factors would allow future research to better link to how maternal stress, including discrimination stress, modulates pregnancy and fetal outcomes [46].

### Strengths and Limitations 

There are some strengths in this study. First, we adopted a prospective cohort study and comprehensively evaluated the association of VD in T1, T2, and T3 with PTB and PROM, and further supplemented the association between maternal VD during delivery and outcomes in the case-control study, which raised the level of evidence for inferences of causation. Second, we considered the increase of 25(OH)D levels during pregnancy and provided the association between the increase of 25(OH)D levels and outcomes. Third, we measured mRNA expression levels of pyroptosis-related factors in maternal PBMC, and placenta and fetal membranes. Fourth, considering that the etiology of PPROM and PTB without PROM may be different, we further differentiated PTB into different types to clarify the association between VD and PTB. Certainly, some limitations should also be mentioned. First, there was the loss of a follow-up with the subjects during pregnancy, which could introduce selection bias. Second, the prevalence of PTB was not very high in Zhoushan and Yiwu, which limited the sample size and the stability of the results. Third, we did not collect the information of medical intervention, so we did not distinguish between spontaneous preterm delivery and iatrogenic preterm delivery. However, when we repeated the analysis among those with vaginal delivery, the results were basically consistent with the overall population. Fourth, the case-control study was not based on the cohort study, which reduced the evidence. Fifth, although HDP and DM were considered as the potential confounding factors, some other important confounding factors (lifestyle, diet, and perinatal complications) were not adjusted.

## 5. Conclusions

In conclusion, 25(OH)D in T3 and the increase of 25(OH)D between T1 and T3 were associated with a lower risk for PTB without PROM but with a higher risk for TPROM. It has been suggested that VD supplementation during pregnancy may be targeted at specific high-risk groups. In maternal PBMC, the mRNA expression of NLRP1 in the TPROM group was higher than that in the term group. No association was found between VD and PPROM or the mRNA expression of pyroptosis-related factors and PPROM. More studies should be conducted to elucidate the association between VD and PTB and PROM and its mechanism.

## Figures and Tables

**Table 1:** Comparison of basic characteristics among groups (*n* = 6381, cohort study).

Variable	Term (*n* = 5274)	TPROM (*n* = 798)	Preterm Birth without PROM (*n* = 237)	PPROM (*n* = 72)	*p*
	Mean ± SD				
**Age, year**	29.31 ± 4.01	29.26 ± 4.04	30.08 ± 4.05	29.97 ± 4.76	0.016
**BMI at first visit, kg/m^2^**	21.24 ± 2.91	21.37 ± 2.93	21.95 ± 3.16	21.94 ± 3.18	0.001
**Weight gain, kg**	12.83 ± 3.82	12.43 ± 3.83	11.43 ± 3.65	11.02 ± 3.17	<0.001
	N (%)				
**Education**					0.025
Primary school and below	783 (14.85)	93 (11.65)	30 (12.66)	11 (15.28)	
High school	485 9.20)	73 (9.15)	28 (11.81)	8 (11.11)	
College and above	3429 (65.02)	512 (64.16)	150 (63.29)	43 (59.72)	
Unknown	577 (10.94)	120 (15.04)	29 (12.24)	10 (13.89)	
**Parity**					0.011
0	3761 (71.31)	612 (76.69)	173 (73.00)	54 (75.00)	
≥1	1513 (28.69)	186 (23.31)	64 (27.00)	18 (25.00)	
DM *					0.195
No	4435 (84.17)	657 (82.33)	196 (83.76)	55 (76.39)	
Yes	834 (15.83)	141 (17.67)	38 (16.24)	17 (23.61)	
**HDP †**					<0.001
No	5023 (95.33)	776 (97.24)	205 (87.61)	71 (98.61)	
Yes	246 (4.67)	22 (2.76)	29 (12.39)	1 (1.39)	
**Delivery mode**					<0.001
Vaginal delivery	2929 (55.54)	600 (75.19)	88 (37.13)	47 (65.28)	
Caesarean section	2345 (44.46)	198 (24.81)	149 (62.87)	25 (34.72)	

TPROM, Term prelabor rupture of membranes; PROM, prelabor rupture of membranes; PPROM, Preterm prelabor rupture of membranes. * DM, diabetes mellitus, including diabetes mellitus before pregnancy and gestational diabetes mellitus. † HDP, hypertension disorder of pregnancy, including chronic hypertension, gestational hypertension, preeclampsia, and eclampsia.

**Table 2:** Comparison of vitamin D levels and gestational age of detection among groups * (cohort study).

Variable	Term	TPROM	Preterm Birth without PROM	PPROM	*p*
	Mean ± SD				
**25(OH)D**, ng/mL					
T1	17.85 ± 8.10	18.01 ± 8.28	17.97 ± 7.70	19.36 ± 9.33	0.508
T2	28.00 ± 11.08	28.24 ± 10.90	28.91 ± 10.85	28.01 ± 11.17	0.859
T3	29.11 ± 12.25	30.50 ± 11.03	25.96 ± 12.15	26.16 ± 11.31	**0.031**
**Gestational age of Vitamin D detection**, week					
T1	11.92 ± 0.92	11.96 ± 0.80	11.83 ± 0.96	12.06 ± 0.75	0.197
T2	24.12 ± 3.42	24.02 ± 3.28	23.58 ± 3.58	24.23 ± 2.67	0.442
T3	33.65 ± 3.72	34.47 ± 3.33	31.86 ± 3.65	32.52 ± 3.89	**<0.001**
	N (%)				
**25(OH)D**, ng/mL					
**T1**					0.575
≥20	1427 (34.16)	223 (36.44)	72 (34.29)	25 (39.68)	
<20	2750 (65.84)	389 (63.56)	138 (65.71)	38 (60.32)	
**T2**					0.742
≥20	1910 (75.05)	338 (74.61)	71 (73.96)	29 (82.86)	
<20	635 (24.95)	115 (25.39)	25 (26.04)	6 (17.14)	
**T3**					**0.037**
≥20	1964 (73.89)	322 (78.73)	27 (61.36)	9 (69.23)	
<20	694 (26.11)	87 (21.27)	17 (38.64)	4 (30.77)	
**Detection season of Vitamin D**					
**T1**					0.329
Summer and autumn	2062 (49.37)	313 (51.14)	113 (53.81)	36 (57.14)	
Winter and spring	2115 (50.63)	299 (48.86)	97 (46.19)	27 (42.86)	
**T2**					0.326
Summer and autumn	1329 (52.22)	247 (54.53)	47 (48.96)	14 (40.00)	
Winter and spring	1216 (47.78)	206 (45.47)	49 (51.04)	21 (60.00)	
**T3**					0.360
Summer and autumn	1293 (48.65)	218 (53.30)	21 (47.73)	6 (46.15)	
Winter and spring	1365 (51.35)	191 (46.70)	23 (52.27)	7 (53.85)	

TPROM, Term prelabor rupture of membranes; PROM, prelabor rupture of membranes; PPROM, preterm prelabor rupture of membranes. T1, first trimester; T2, second trimester; T3, third trimester. * T1, N = 5062; T2, N = 3129; T3, N = 3124.

**Table 3:** Association between vitamin D status during pregnancy and delivery outcomes (cohort study).

25(OH)D, ng/mL	Term	TPROM			Preterm Birth without PROM			PPROM		
	N (%)	N (%)	OR (95% CI)	*p*	N (%)	OR (95% CI)	*p*	N (%)	OR (95% CI)	*p*
	Model 1 ^a^									
**T1**	4177	612	1.00 (0.99–1.01)	0.6539	210	1.00 (0.98–1.02)	0.8344	63	1.02 (0.99–1.05)	0.1422
≥20	1427 (81.7)	223 (12.8)	ref.	-	72 (4.1)	ref.	-	25 (1.4)	ref.	-
<20	2750 (83.0)	389 (11.7)	0.91 (0.76–1.08)	0.2688	138 (4.2)	0.99 (0.74–1.33)	0.9694	38 (1.1)	0.79 (0.47–1.31)	0.3594
**T2**	2545	453	1.00 (0.99–1.01)	0.6715	96	1.01 (0.99–1.03)	0.4281	35	1.00 (0.97–1.03)	0.9986
≥20	1910 (81.3)	338 (14.4)	ref.	-	71 (3.0)	ref.	-	29 (1.2)	ref.	-
<20	635 (81.3)	115 (14.7)	1.02 (0.81–1.29)	0.8437	25 (3.2)	1.06 (0.67–1.69)	0.8086	6 (0.8)	0.62 (0.26–1.51)	0.2928
**T3**	2658	409	1.01 (1.00–1.02)	**0.0307**	44	0.98 (0.95–1.00)	0.0870	13	0.98 (0.93–1.03)	0.3797
≥20	1964 (84.6)	322 (13.9)	ref.	-	27 (1.2)	ref.	-	9 (0.4)	ref.	-
<20	694 (86.5)	87 (10.8)	0.76 (0.59–0.98)	**0.0370**	17 (2.1)	1.78 (0.97–3.29)	0.0648	4 (0.5)	1.26 (0.39–4.10)	0.7036
	Model 2 ^b^									
**T1**	4177	612	1.01 (0.99–1.02)	0.3456	210	1.00 (0.98–1.02)	0.9850	63	1.02 (0.99–1.05)	0.1395
≥20	1427 (81.7)	223 (12.8)	ref.	-	72 (4.1)	ref.	-	25(1.4)	ref.	-
<20	2750 (83.0)	389 (11.7)	0.87 (0.73–1.05)	0.1418	138 (4.2)	1.04 (0.77–1.40)	0.8077	38 (1.1)	0.79 (0.47–1.33)	0.3786
**T2**	2545	453	1.00 (0.99–1.01)	0.6751	96	1.01 (0.99–1.03)	0.2398	35	1.00 (0.97–1.04)	0.8332
≥20	1910 (81.3)	338 (14.4)	ref.	-	71 (3.0)	ref.	-	29 (1.2)	ref.	-
<20	635 (81.3)	115 (14.7)	1.02 (0.81–1.29)	0.8353	25 (3.2)	1.03 (0.64–1.65)	0.9134	6 (0.8)	0.59 (0.24–1.44)	0.2455
**T3**	2658	409	1.01 (1.00–1.02)	**0.0310**	44	0.98 (0.95–1.00)	0.0806	13	0.98 (0.94–1.03)	0.4860
≥20	1964 (84.6)	322 (13.9)	ref.	-	27 (1.2)	ref.	-	9 (0.4)	ref.	-
<20	694 (86.5)	87 (10.8)	0.76 (0.59–0.98)	**0.0373**	17 (2.1)	1.90 (1.02–3.55)	**0.0446**	4 (0.5)	1.07 (0.33–3.51)	0.9109
	Model 3 ^c^									
**T1**	4177	612	1.00 (0.99–1.02)	0.3690	210	1.00 (0.98–1.02)	0.7261	63	1.02 (0.99–1.05)	0.2149
≥20	1427 (81.7)	223 (12.8)	ref.	-	72 (4.1)	ref.	-	25 (1.4)	ref.	-
<20	2750 (83.0)	389 (11.7)	0.89 (0.74–1.07)	0.2022	138 (4.2)	1.08 (0.80–1.47)	0.6148	38 (1.1)	0.85 (0.50–1.44)	0.5509
**T2**	2545	453	1.00 (0.99–1.01)	0.7113	96	1.02 (1.00–1.04)	0.0882	35	1.01 (0.97–1.04)	0.7179
≥20	1910 (81.3)	338 (14.4)	ref.	-	71 (3.0)	ref.	-	29 (1.2)	ref.	-
<20	635 (81.3)	115 (14.7)	1.05 (0.82–1.34)	0.7182	25 (3.2)	0.91 (0.55–1.51)	0.7104	6 (0.8)	0.53 (0.21–1.34)	0.1791
**T3**	2658	409	1.00 (0.99–1.01)	0.5635	44	0.99 (0.96–1.02)	0.3634	13	0.99 (0.94–1.04)	0.6188
≥20	1964 (84.6)	322 (13.9)	ref.	-	27 (1.2)	ref.	-	9 (0.4)	ref.	-
<20	694 (86.5)	87 (10.8)	0.92 (0.70–1.20)	0.5318	17 (2.1)	1.50 (0.76–2.93)	0.2405	4 (0.5)	0.94 (0.26–3.39)	0.9289

TPROM, Term prelabor rupture of membranes; PROM, prelabor rupture of membranes; PPROM, preterm prelabor rupture of membranes; T1, first trimester; T2, second trimester; T3, third trimester. ^a^ Model 1 was crude model. ^b^ Model 2 was adjusted for BMI at first visit, age, education, parity, and hypertension disorder of pregnancy. ^c^ Model 3 was adjusted for variables in Model 2 and the gestational age of vitamin D detection, season of vitamin D detection, and delivery mode.

**Table 4:** Association of vitamin D level changes between T1 and T3 with delivery outcomes (cohort study).

25(OH)D, ng/mL	Term	TPROM			Preterm Birth without PROM			PPROM		
	N (%)	N (%)	OR (95% CI)	*p*	N (%)	OR (95% CI)	*p*	N (%)	OR (95% CI)	*p*
	Model 1 ^a^									
**Change of 25(OH)D**										
≥10.8	1022 (83.8)	181 (14.8)	ref.	-	10 (0.8)	ref.	-	7 (0.6)	ref.	-
<10.8	1054 (86.3)	131 (10.7)	0.70 (0.55–0.89)	**0.0039**	30 (2.5)	2.91 (1.41–5.98)	**0.0037**	6 (0.5)	0.83 (0.28–2.48)	0.7403
	Model 2 ^b^									
**Change of 25(OH)D**										
≥10.8	1022 (83.8)	181 (14.8)	ref.	-	10 (0.8)	ref.	-	7 (0.6)	ref.	-
<10.8	1054 (86.3)	131 (10.7)	0.67 (0.52–0.86)	**0.0018**	30 (2.5)	2.89 (1.37–6.12)	**0.0054**	6 (0.5)	0.87 (0.28–2.69)	0.8034
	Model 3 ^c^									
**Change of 25(OH)D**										
≥10.8	1022 (83.8)	181 (14.8)	ref.	-	10 (0.8)	ref.	-	7 (0.6)	ref.	-
<10.8	1054 (86.3)	131 (10.7)	0.73 (0.56–0.96)	**0.0235**	30 (2.5)	2.32 (1.07–5.06)	**0.0337**	6 (0.5)	0.71 (0.21–2.39)	0.5843

TPROM, Term prelabor rupture of membranes; PROM, prelabor rupture of membranes; PPROM, preterm prelabor rupture of membranes. ^a^ Model 1 was crude model. ^b^ Model 2 was adjusted for BMI at first visit, age, education, parity, hypertension disorder of pregnancy, and baseline vitamin D. ^c^ Model 3 was adjusted for variables in Model 2 and gestational age of vitamin D detection, season of vitamin D detection, and delivery mode.

**Table 5:** Comparison of basic characteristics and VD levels when awaiting delivery between groups (case-control study).

Variable	Term (N = 37) ^†^	PPROM (N = 37)	*p*	Term (N = 15) ^‡^	Preterm without PROM (N = 16)	*p*	Term (N = 55) ^§^	TPROM (N = 58)	*p*
	N (%)			N (%)			N (%)		
**Gravidity**			0.913			0.547			0.047
1	16 (43.2)	17 (45.9)		3 (20.0)	3 (18.8)		21 (38.2)	24 (41.4)	
2	11 (29.7)	9 (24.3)		10 (66.7)	8 (50.0)		21 (38.2)	11 (19.0)	
≥3	10 (27.0)	11 (29.7)		2 (13.3)	5 (31.2)		13 (23.6)	23 (39.7)	
**Parity**			0.813			1.000			0.259
0	21 (56.8)	23 (62.2)		5 (33.3)	5 (31.2)		28 (50.9)	36 (62.1)	
≥1	16 (43.2)	14 (37.8)		10 (66.7)	11 (68.8)		27 (49.1)	22 (37.9)	
**Delivery mode**			1.000			0.716			0.001
Vaginal delivery	20 (54.1)	21 (56.8)		5 (33.3)	7 (43.8)		28 (50.9)	47 (81.0)	
Caesarean delivery	17 (45.9)	16 (43.2)		10 (66.7)	9 (56.2)		27 (49.1)	11 (19.0)	
**VD deficiency**			1.000			0.101 *			0.304
No	24 (64.9)	25 (67.6)		15 (100.0)	12 (75.0)		42 (76.4)	39 (67.2)	
Yes	13 (35.1)	12 (32.4)		0 (0.0)	4 (25.0)		13 (23.6)	19 (32.8)	
	Mean ± SD			Mean ± SD			Mean ± SD		
**Delivery week**, week	40.04 ± 0.95	34.91 ± 1.78	<0.001	39.48 ± 0.79	34.94 ± 2.05	<0.001	39.84 ± 0.94	39.15 ± 1.30	0.001
**Maternal age**, years	28.70 ± 3.81	28.51 ± 3.78	0.831	29.60 ± 4.05	29.75 ± 3.28	0.910	28.96 ± 3.77	29.00 ± 4.75	0.964
**Pre-pregnancy BMI**, kg/m^2^	21.47 ± 3.01	23.93 ± 3.44	0.089	22.55 ± 4.18	21.76 ± 4.17	0.603	22.19 ± 3.41	21.66 ± 3.68	0.423
**25(OH)D**, ng/mL	27.02 ± 10.46	25.79 ± 5.77	0.620	39.31 ± 14.59	28.47 ± 15.20	0.052	30.66 ± 12.62	26.46 ± 14.20	0.100

TPROM, term prelabor rupture of membranes; PROM, prelabor rupture of membranes; PPROM, preterm prelabor rupture of membranes; VD, vitamin D. Term † and term ‡ were two different term control groups; Term § includes term † and term ‡; * Compared with Fisher’s exact test.

## Data Availability

The data presented in this study are available on request from the corresponding author. The data are not publicly available because they contain information that could compromise the privacy of research participants.

## Supplementary Materials

The following supporting information can be downloaded at: https://www.mdpi.com/article/10.3390/nu15153423/s1, Figure S1: The flow chart of case-control study; Table S1: The sequence of primers; Table S2: Association between vitamin D status during pregnancy and delivery outcomes (among Vaginal delivery); Table S3: Association of vitamin D levels change between T1 and T2 with and delivery outcomes; Table S4: Association of vitamin D levels change between T2 and T3 with and delivery outcomes; Table S5: Association with vitamin D levels when awaiting delivery and delivery outcomes (case-control study); Table S6: Comparison of basic characteristics, VD levels, and mRNA expression in placenta between groups; Table S7: Comparison of basic characteristics, VD levels, and mRNA expression in fetal membrane between groups.
