# Supplementary material for: The Association of Vitamin D during Pregnancy and mRNA Expression Levels of Inflammatory Factors with Preterm Birth and Prelabor Rupture of Membranes
Source: Nutrients. 2023 Aug 2;15(15):3423. doi: 10.3390/nu15153423 (PMC10421124; doi:10.3390/nu15153423)
Supplement: Supplementary file 1 [file nutrients-15-03423-s001.zip › nutrients-2490235-supplementary.pdf]

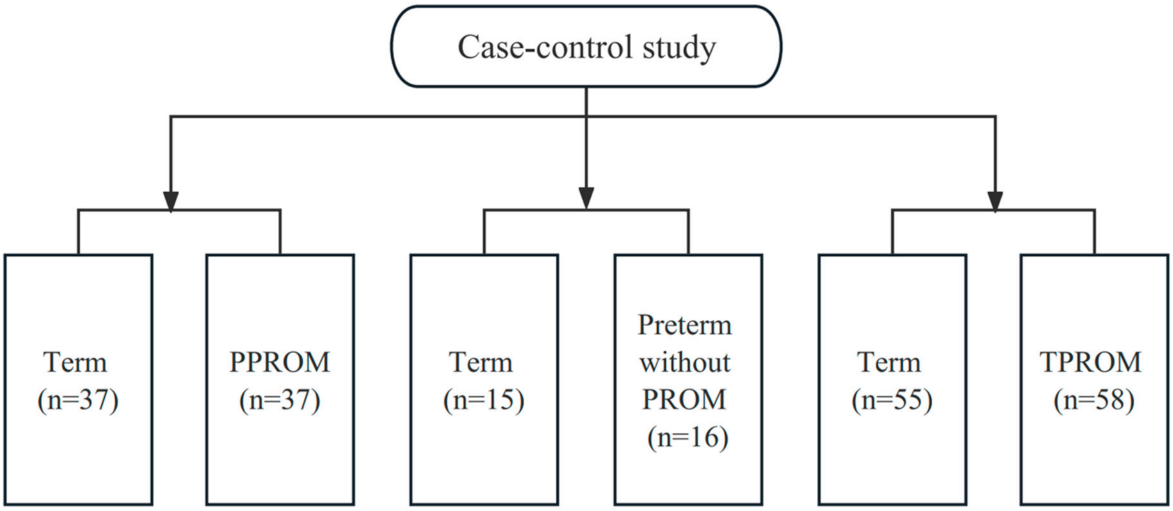

Supplementary Figure S1. The flow chart of case-control study.

Supplementary Table S1. The sequence of Primers

Primers	Sequence(5' to 3')
H-GAPDH-F	ATCACTGCCACCCAGAAGAC
H-GAPDH-R	TTTCTAGACGGCAGGTCAGG
H-NLRP1-F	CCACAACCCTCTGTCTACATTAC
H-NLRP1-R	GCCCCATCTAACCCATGCTTC
H-NLRP3-F	GATCTTCGCTGCGATCAACAG
H-NLRP3-R	CGTGCATTATCTGAACCCCAC
H-NLRC4-F	CCAGTCCCCTCACCATAGAAG
H-NLRC4-R	ACCCAAGCTGTCAGTCAGACC
H-AIM2-F	TGGCAAAACGTCTTCAGGAGG
H-AIM2-R	AGCTTGACTTAGTGGCTTTGG
H-ASC-F	AACCCAAGCAAGATGCGGAAG
H-ASC-R	TTAGGGCCTGGAGGAGCAAG
H-Gasdermin D-F	GAGTGTGGCCTAGAGCTGG
H-Gasdermin D-R	GGCTCAGTCCTGATAGCAGTG
H-Caspase-1-F	GCCTGTTCCTGTGATGTGGAG
H-Caspase-1-R	TGCCCACAGACATTCATACAGTTTC
H-IL-1β-F	TTCGACACATGGGATAACGAGG
H-IL-1β-R	TTTTTGCTGTGAGTCCCGGAG
H-IL-18-F	TCTTCATTGACCAAGGAAATCGG
H-IL-18-R	TCCGGGGTGCATTATCTCTAC

Supplementary Table S2. Association between vitamin D status during pregnancy and delivery outcomes (among Vaginal delivery)

25(OH)D, ng/mL	TPROM		Preterm birth without PROM		PPROM	
	OR (95% CI)	*P*	OR (95% CI)	*P*	OR (95% CI)	*P*
			Model 1a			
T1						
≥20	ref.	-	ref.	-	ref.	-
<20	0.90 (0.73 - 1.11)	0.3389	1.18 (0.73 - 1.92)	0.5019	0.70 (0.37 - 1.31)	0.2685
T2						
≥20	ref.	-	ref.	-	ref.	-
<20	1.06 (0.81 - 1.40)	0.6644	0.66 (0.25 - 1.75)	0.4048	0.50 (0.15 - 1.70)	0.269
T3						
≥20	ref.	-	ref.	-	ref.	-
<20	0.86 (0.64 - 1.16)	0.3319	3.13 (1.23 - 7.95)	0.0163	2.09 (0.59 - 7.45)	0.2551
			Model 2b			
T1						
≥20	ref.	-	ref.	-	ref.	-
<20	0.91 (0.73 - 1.13)	0.3813	1.18 (0.72 - 1.93)	0.5045	0.76 (0.40 - 1.43)	0.3919
T2						
≥20	ref.	-	ref.	-	ref.	-
<20	1.06 (0.80 - 1.40)	0.6843	0.60 (0.22 - 1.62)	0.3129	0.47 (0.14 - 1.60)	0.2257
T3						
≥20	ref.	-	ref.	-	ref.	-
<20	0.88 (0.65 - 1.19)	0.3975	2.82 (1.09 - 7.25)	0.0320	1.92 (0.53 - 6.93)	0.3173
			Model 3[c]			
T1						

≥20	ref.	-	ref.	-	ref.	-
<20	0.93 (0.75 - 1.15)	0.5066	1.25 (0.76 - 2.07)	0.3751	0.86 (0.45 - 1.64)	0.6409
T2						
≥20	ref.	-	ref.	-	ref.	-
<20	1.05 (0.79 - 1.41)	0.7298	0.69 (0.25 - 1.92)	0.4796	0.44 (0.12 - 1.56)	0.2021
T3						
≥20	ref.	-	ref.	-	ref.	-
<20	0.98 (0.71 - 1.34)	0.8881	2.72 (0.99 - 7.44)	0.0514	1.46 (0.37 - 5.75)	0.5924

TPROM, Term prelabor rupture of membranes; PROM, prelabor rupture of membranes; PPROM, Preterm prelabor rupture of membranes; T1, First trimester; T2, Second trimester; T3, Third trimester

[a]Model1 was crude model

[b]Model2 was adjusted for BMI at first visit, age, education, parity and Hypertension disorder of pregnancy

[c]Model3 was adjusted for variables in Model2 and gestational age of Vitamin D detection, season of Vitamin D detection.

Supplementary Table S3. Association of vitamin D levels change between T1 and T2 with and delivery outcomes

25(OH)D, ng/mL	Term	TPROM			Preterm birth without PROM			PPROM		
	N (%)	N (%)	OR (95% CI)	*P*	N (%)	OR (95% CI)	*P*	N (%)	OR (95% CI)	*P*
					Model 1[a]					
**change of 25(OH)D**										
≥10.4	848(80.1)	160(15.1)	ref.	-	41(3.9)	ref.	-	10(0.9)	ref.	-
<10.4	861(81.5)	148(14.0)	0.91 (0.71 - 1.16)	0.4545	31(2.9)	0.74 (0.46 - 1.20)	0.2243	16(1.5)	1.58 (0.71 - 3.50)	0.2614
					Model 2[b]					
**change of 25(OH)D**										
≥10.4	848(80.1)	160(15.1)	ref.	-	41(3.9)	ref.	-	10(0.9)	ref.	-
<10.4	861(81.5)	148(14.0)	0.92 (0.71 - 1.20)	0.5489	31(2.9)	0.58 (0.35 - 0.97)	0.0384	16(1.5)	1.18 (0.51 - 2.74)	0.6998
					Model 3[c]					
**change of 25(OH)D**										
≥10.4	848(80.1)	160(15.1)	ref.	-	41(3.9)	ref.	-	10(0.9)	ref.	-
<10.4	861(81.5)	148(14.0)	0.95 (0.73 - 1.23)	0.7004	31(2.9)	0.58 (0.35 - 0.98)	0.0414	16(1.5)	1.19 (0.51 - 2.79)	0.6841

TPROM, Term prelabor rupture of membranes; PROM, prelabor rupture of membranes; PPROM, Preterm prelabor rupture of membranes; T1, First trimester; T2, Second trimester

[a]Model1 was crude model

[b]Model2 was adjusted for BMI at first visit, age, education, parity, Hypertension disorder of pregnancy and baseline Vitamin D

[c]Model3 was adjusted for variables in Model2 and gestational age of Vitamin D detection, season of Vitamin D detection and delivery mode.

Supplementary Table S4. Association of vitamin D levels change between T2 and T3 with and delivery outcomes

25(OH)D, ng/mL	Term	TPROM			Preterm birth without PROM			PPROM		
	N (%)	N (%)	OR (95% CI)	*P*	N (%)	OR (95% CI)	*P*	N (%)	OR (95% CI)	*P*
					Model 1[a]					
**change of 25(OH)D**										
≥3.8	473(80.7)	109(18.6)	ref.	-	3(0.5)	ref.	-	1(0.2)	ref.	-
<3.8	580(83.9)	99(14.3)	0.74 (0.55 - 1.00)	0.0484	10(1.4)	2.72 (0.74 - 9.94)	0.1304	2(0.3)	1.63 (0.15 - 17.99)	0.6910
					Model 2[b]					
**change of 25(OH)D**										
≥3.8	473(80.7)	109(18.6)	ref.	-	3(0.5)	ref.	-	1(0.2)	ref.	-
<3.8	580(83.9)	99(14.3)	0.76 (0.56 - 1.03)	0.0778	10(1.4)	2.78 (0.74 - 10.54)	0.1318	2(0.3)	1.81 (0.16 - 20.44)	0.6322
					Model 3[c]					
**change of 25(OH)D**										
≥3.8	473(80.7)	109(18.6)	ref.	-	3(0.5)	ref.	-	1(0.2)	ref.	-
<3.8	580(83.9)	99(14.3)	0.77 (0.56 - 1.05)	0.0988	10(1.4)	2.54 (0.65 - 9.96)	0.1807	2(0.3)	23.05 (0.14 - 3713.63)	0.2263

TPROM, Term prelabor rupture of membranes; PROM, prelabor rupture of membranes; PPROM, Preterm prelabor rupture of membranes; T2, Second trimester; T3, Third trimester

[a]Model1 was crude model

[b]Model2 was adjusted for BMI at first visit, age, education, parity, Hypertension disorder of pregnancy and baseline Vitamin D

[c]Model3 was adjusted for variables in Model2 and gestational age of Vitamin D detection, season of Vitamin D detection and delivery mode.

Supplementary Table S5. Association with vitamin D levels when awaiting delivery and delivery outcomes (case-control study)

25(OH)D, ng/mL	Term	TPROM			Preterm birth without PROM			PPROM		
	N (%)	N (%)	OR (95% CI)	*P*	N (%)	OR (95% CI)	*P*	N (%)	OR (95% CI)	*P*
					Model 1[a]					
**25(OH)D**	65	58	0.98 (0.95 - 1.00)	0.1035	16	0.99 (0.95 - 1.03)	0.5944	37	0.97 (0.94 - 1.01)	0.0981
≥20	42(35.6)	39(33.1)	ref.	-	12(10.2)	ref.	-	25(21.2)	ref.	-
<20	13(27.1)	19(39.6)	1.57 (0.69 - 3.61)	0.2837	4(8.3)	1.08 (0.30 - 3.92)	0.9104	12(25.0)	1.55 (0.61 - 3.92)	0.3540
					Model 2[b]					
**25(OH)D**	65	58	0.98 (0.95 - 1.01)	0.2439	16	0.97 (0.92 - 1.02)	0.2551	37	0.97 (0.94 - 1.01)	0.1879
≥20	42(35.6)	39(33.1)	ref.	-	12(10.2)	ref.	-	25(21.2)	ref.	-
<20	13(27.1)	19(39.6)	1.33 (0.52 - 3.44)	0.5549	4(8.3)	1.66 (0.33 - 8.19)	0.5365	12(25.0)	1.19 (0.42 - 3.40)	0.7422

TPROM, Term prelabor rupture of membranes; PROM, prelabor rupture of membranes; PPROM, Preterm prelabor rupture of membranes; T1, First trimester; T2, Second trimester

[a]Model1 was crude model

[b]Model2 was adjusted for pre-pregnancy BMI, age, parity, Diabetes, Hypertension disorder of pregnancy, delivery mode and year of Vitamin D detection.

Supplementary Table S6. Comparison of basic characteristics, VD levels and mRNA expression in placenta between groups

Variables	Term (N=30)†	PPROM (N=27)	*P*	Term (N=10)‡	Preterm without PROM (N=12)	*P*	Term (N=46)§	TPROM (N=45)	*P*
	Mean ± SD			Mean ± SD			Mean ± SD		
**Delivery week**, week	39.95 ± 0.99	35.13 ± 1.63	<0.001	39.47 ± 0.87	34.57 ± 2.16	<0.001	39.88 ± 0.99	39.19 ± 1.27	0.005
**Maternal age**, years	28.67 ± 4.03	29.07 ± 3.58	0.690	29.40 ± 3.20	29.08 ± 3.37	0.825	28.70 ± 3.66	28.38 ± 4.26	0.703
**pre-pregnancy BMI**, kg/m^2	22.44 ± 3.28	22.79 ± 3.48	0.699	23.45 ± 4.79	22.18 ± 4.53	0.529	22.50 ± 3.62	21.53 ± 4.02	0.229
	N (%)			N (%)			N (%)		
**Gravidity**			0.944			0.267			0.288
1	12 (40.0)	11 (40.7)		2 (20.0)	3 (25.0)		18 (39.1)	20 (44.4)	
2	8 (26.7)	6 (22.2)		8 (80.0)	6 (50.0)		17 (37.0)	10 (22.2)	
≥3	10 (33.3)	10 (37.0)		0 (0.0)	3 (25.0)		11 (23.9)	15 (33.3)	
**Parity**			1.000			1.000			0.290
0	16 (53.3)	15 (55.6)		3 (30.0)	4 (33.3)		24 (52.2)	29 (64.4)	
≥1	14 (46.7)	12 (44.4)		7 (70.0)	8 (66.7)		22 (47.8)	16 (35.6)	
**Delivery mode**			0.793			0.231			0.001
Vaginal delivery	17 (56.7)	14 (51.9)		3 (30.0)	7 (58.3)		24 (52.2)	38 (84.4)	
Cesarean delivery	13 (43.3)	13 (48.1)		7 (70.0)	5 (41.7)		22 (47.8)	7 (15.6)	
				mRNA expression relative to GAPDH (median [IQR]) §					
**ASC**	0.84 [0.58, 1.60]	1.02 [0.53, 1.76]	0.724	0.80 [0.61, 2.32]	1.06 [0.50, 1.79]	1.000	0.97 [0.53, 1.53]	1.08 [0.64, 1.92]	0.446

**Caspase 1**	1.10 [0.54, 2.14]	0.87 [0.49, 1.32]	0.415	1.02 [0.31, 2.69]	1.51 [0.54, 3.39]	0.843	0.99 [0.44, 2.41]	1.57 [0.28, 4.85]	0.818
**Gasdermin D**	1.17 [0.50, 2.26]	0.65 [0.46, 1.34]	0.137	0.81 [0.40, 2.73]	1.95 [0.82, 4.15]	0.429	1.10 [0.59, 2.58]	1.16 [0.44, 2.73]	0.709
**IL-18**	0.94 [0.54, 2.08]	0.87 [0.42, 1.44]	0.355	0.70 [0.56, 1.82]	1.29 [0.21, 2.97]	0.847	1.00 [0.30, 2.19]	0.67 [0.48, 2.53]	0.690
**IL-1β**	0.97 [0.51, 1.77]	0.85 [0.43, 1.75]	0.936	0.67 [0.52, 1.84]	0.78 [0.17, 3.58]	0.692	0.86 [0.49, 2.09]	1.01 [0.49, 2.54]	0.460
**NLRP1**	1.01 [0.58, 1.97]	0.80 [0.52, 1.32]	0.283	1.07 [0.53, 2.49]	1.73 [0.87, 3.03]	0.360	1.13 [0.57, 2.46]	1.26 [0.30, 2.53]	0.737
**NLRP3**	0.81 [0.53, 1.98]	0.72 [0.17, 1.40]	0.240	0.48 [0.29, 14.59]	2.37 [0.24, 11.96]	0.817	0.72 [0.45, 2.86]	0.86 [0.25, 1.96]	0.656

TPROM, Term prelabor rupture of membranes; PROM, prelabor rupture of membranes; PPROM, Preterm prelabor rupture of membranes; VD, Vitamin D

Term† and Term‡ were two different term control groups ;Term§ includes Term† and Term‡; *Compared with fisher's exact test; §Compared with wilcox-test.

Supplementary Table S7. Comparison of basic characteristics, VD levels and mRNA expression in fetal membrane between groups

**Variables**	Term (N=30)†	PPROM (N=28)	*P*	Term (N=11)‡	Preterm without PROM (N=13)	*P*	Term (N=46)§	TPROM (N=45)	*P*
**Delivery week**, week	40.04 ± 0.99	35.10 ± 1.63	<0.001	39.53 ± 0.85	34.73 ± 2.15	<0.001	39.90 ± 0.99	39.27 ± 1.26	0.009
**Maternal age**, years	28.70 ± 3.91	28.79 ± 3.97	0.934	29.36 ± 3.04	30.31 ± 2.56	0.418	28.63 ± 3.62	28.47 ± 4.28	0.844
**pre-pregnancy BMI**, kg/m^2	22.16 ± 3.34	22.86 ± 3.36	0.430	23.14 ± 4.66	22.55 ± 4.22	0.745	22.29 ± 3.66	21.88 ± 3.96	0.610
**Gravidity**			0.801			0.193			0.270
1	11 (36.7)	11 (39.3)		3 (27.3)	3 (23.1)		18 (39.1)	19 (42.2)	
2	10 (33.3)	7 (25.0)		8 (72.7)	6 (46.2)		18 (39.1)	11 (24.4)	
≥3	9 (30.0)	10 (35.7)		0 (0.0)	4 (30.8)		10 (21.7)	15 (33.3)	
**Parity**			0.798			1.000			0.528
0	16 (53.3)	16 (57.1)		4 (36.4)	5 (38.5)		24 (52.2)	27 (60.0)	
≥1	14 (46.7)	12 (42.9)		7 (63.6)	8 (61.5)		22 (47.8)	18 (40.0)	
**Delivery mode**			1.000			0.423			0.001
Vaginal delivery	18 (60.0)	16 (57.1)		3 (27.3)	6 (46.2)		24 (52.2)	38 (84.4)	
Cesarean delivery	12 (40.0)	12 (42.9)		8 (72.7)	7 (53.8)		22 (47.8)	7 (15.6)	
	mRNA expression relative to GAPDH (median [IQR])*								
**ASC**	0.96 [0.61, 1.70]	1.32 [0.57, 1.97]	0.876	0.82 [0.76, 1.56]	1.07 [0.70, 1.45]	0.885	1.11 [0.65, 1.71]	1.21 [0.72, 2.00]	0.557
**Caspase 1**	1.09 [0.61, 1.66]	1.05 [0.66, 1.68]	0.950	0.99 [0.63, 1.77]	1.50 [0.66, 2.09]	0.750	1.09 [0.55, 2.05]	1.26 [0.77, 1.72]	0.573
**Gasdermin D**	1.29 [0.69, 1.53]	1.17 [0.59, 1.66]	0.815	1.22 [0.49, 1.82]	1.26 [1.09, 1.90]	0.543	1.19 [0.67, 1.53]	1.10 [0.72, 1.91]	0.751

**IL-18**	1.19 [0.69, 1.62]	1.22 [0.63, 1.64]	0.864	1.25 [0.60, 1.60]	1.04 [0.60, 3.58]	0.469	1.24 [0.53, 2.14]	0.85 [0.53, 1.65]	0.465
**IL-1β**	1.19 [0.75, 1.49]	0.74 [0.45, 1.39]	0.087	1.25 [0.70, 1.57]	1.19 [0.78, 1.62]	0.885	1.26 [0.68, 1.72]	0.93 [0.53, 1.89]	0.357
**NLRP1**	1.05 [0.59, 2.43]	0.68 [0.45, 1.26]	0.181	0.99 [0.59, 2.01]	1.04 [0.69, 2.53]	0.582	0.90 [0.48, 2.41]	0.87 [0.46, 1.45]	0.146
**NLRP3**	1.05 [0.68, 1.65]	0.83 [0.46, 1.55]	0.237	0.93 [0.78, 1.39]	0.97 [0.54, 1.31]	0.706	0.94 [0.56, 1.88]	0.83 [0.42, 1.73]	0.210

TPROM, Term prelabor rupture of membranes; PROM, prelabor rupture of membranes; PPROM, Preterm prelabor rupture of membranes; VD, Vitamin D

Term† and Term‡ were two different term control groups ;Term§ includes Term† and Term‡；

*Compared with wilcox-test.
